# Opportunistic predation of birds by breeding Herring Gulls (*Larus argentatus*)

**DOI:** 10.1371/journal.pone.0239180

**Published:** 2020-10-15

**Authors:** Laura R. Ingraham, Stephen A. Oswald, Eunice Son, Jennifer M. Arnold

**Affiliations:** Division of Science, Pennsylvania State University, Reading, PA, United States of America; University of Jyväskylä, FINLAND

## Abstract

Dietary specialization, exploiting a small fraction of available food resources, is commonly reported for gulls and skuas. Predation of birds by these species is usually considered a specialist strategy employed by the minority of the population but non-specialists also predate birds and may actually have a greater impact on the prey species. To date, most studies have focused on predatory bird-specialists, down-playing the possible importance of opportunistic predation by non-specialists. We addressed this by studying diet (regurgitated pellets and prey remains) and behavior of breeding Herring Gulls (*Larus argentatus*) over three summers at Gull Island, a mixed-species breeding colony in Lake Ontario. One-third of all pellets analyzed contained bird remains, primarily the most numerous breeding bird: Ring-billed Gull (*L*. *delawarensis*) chicks (51%) and adults (36%). Although all but one pair of Herring Gulls ate birds, all pairs maintained broad and mostly similar diets, with birds accounting for at most one-third of prey. Behavior also indicated that Herring Gulls at Gull Island were not predatory bird-specialists because predation was too infrequent to meet energetic requirements, was largely unsuccessful and was only ever observed when Ring-billed Gulls strayed into Herring Gull breeding territories. Instead, bird predation appeared mainly opportunistic, increasing with seasonal availability, access to shoreline, proximity to nesting Ring-billed Gulls and breeding territory size. Compared with predatory specialist Herring Gulls in the same region, individuals that predated birds at Gull Island did not display specialist behaviors and killed six times fewer birds (0.1–0.4 per day, on average) but were over 20 times more numerous (98% of the population versus 4%). Thus, our results indicate that opportunistic predation by non-specialists may have important consequences for prey species. Since opportunistic predation cannot be effectively managed using techniques widely advocated for specialist predators, it is essential to investigate cause of predation by large gulls prior to lethal management.

## Introduction

Specialist predators have a narrow dietary niche, exploiting a small fraction of available resources, often more efficiently than generalists that utilize a wider range of prey [1]. There has been growing interest in the causes and consequences of individual specialization, especially in diet studies [2–6]. Individual dietary specialization has been widely reported for seabirds, most commonly among Charadriiform species, of which large gulls *Larus* spp. or skuas *Stercorarius* spp., account for greater than 60% of records [7]. These two genera of birds often occupy similar upper trophic niches at mixed-species colonies, where they scavenge, feed on marine and terrestrial prey or predate other birds [8,9]. Predation of birds is commonly described as one specialist strategy employed by a small subset of individual large gulls and skuas, comprising from ~2–4% [10,11] to at most 14–20% [12–14] of individuals within the population. Non-specialists, however, may also predate birds and, because they comprise the majority of the breeding population, their overall impact on the prey species may be greater than that of specialist individuals [15].

Incorrect classification of specialists has important empirical repercussions because lethal control of large gulls is strongly advocated to control specialist individuals that predate conservation-important bird species [16,17]. Behavioral metrics that could be used concurrently to improve identification of predatory bird-specialists include: predation outside of a bird’s own breeding territory [18,19], use of specialized hunting behaviors [12,20,21], and elongation of breeding territories into feeding territories [12,22] or defense of separate feeding territories [10,14,15]. Lethal control methods may become ineffectual and ethically-questionable in cases where non-specialists are having a much greater overall impact. Thus, understanding the degree to which predation is opportunistic and the factors that promote predation of birds by non-specialists is of considerable importance.

To date, most studies of gulls and skuas have focused on predatory bird-specialists [10–12,14,17], as a consequence down-playing the importance of non-bird-specialists (throughout the paper we use the term “non-specialists” in reference to bird prey, acknowledging that these individuals may specialize on other prey). For most gulls and skuas, however, there likely exists a continuous gradient of exploitation of dietary items across the population [23], with individuals specializing to different degrees and also opportunistically exploiting other food resources. In most previous work, predatory bird-specialists are identified based on dietary threshold (e.g. birds comprising > 75% of dietary items [13]). Such categorization may either ignore the impact of non-bird-specialists (e.g. [14]) or divide it among other dietary specialisms (e.g. [13]), unintentionally obscuring the large overall impact of non-specialists on prey populations.

Here, we investigate predation of birds by breeding Herring Gulls (*Larus argentatus*) within a large Ring-billed Gull (*L*. *delawarensis*) breeding colony in Lake Ontario. We focus at the within-colony scale because variation in diet between individuals and pairs within colonies [7,13] suggests that factors influencing diet act at this level. Previous studies in this region have concluded that the majority of predation by Herring Gulls at Ring-billed Gull colonies occurs through the action of predatory bird-specialists [11]. However, prior to our study, we observed extensive seasonal variation in the exploitation of Ring-billed Gulls as prey by Herring Gulls (as indicated by prey remains at breeding territories) which challenged this, suggesting instead that predation could be largely opportunistic. This motivated us to investigate the factors driving predation of birds by Herring Gulls at our colony to determine the degree to which opportunism can be an important component of bird predation by large gulls. Accordingly, we first quantified the level of Herring Gull dietary specialism through analyses of diet and behavior, and then examined factors associated with the frequency of bird predation by non-specialist individuals. If predation of birds is largely opportunistic, we predict that all study birds will predate birds and factors that increase opportunities for such predation (e.g. proximity to prey, availability of vulnerable chicks) will be most strongly associated with its prevalence.

## Methods

### Study site and field methods

We undertook a series of studies of Herring Gulls nesting at Gull Island, Presqu’ile Provincial Park, Ontario (43.98° N, 77.74° W) between May and July in 2009, 2015 and 2016. Gull Island is a 7.3 hectare, vegetated (but essentially treeless), low-lying, limestone island in Lake Ontario, ~300 m from the mainland. Approximately 100 pairs of Herring Gulls nested on the island in each year of the study. The majority of breeding territories were around the edge of the island and had lake access via a gravel beach but some also bred among other waterbirds in the vegetated island interior or on their own in a small colony (~30 nests) at the southeast end. As of 2016, this island also supported ~30,000 pairs of Ring-billed Gull, ~4,000 pairs of Double-crested Cormorant (*Phalacrocorax auritus*), ~600 pairs of Caspian Tern (*Hydroprogne caspia*), and ~100 pairs of Common Tern (*Sterna hirundo*).

All study activities were approved by Penn State University’s Institutional Animal Care and Use Committee (protocols #28103 and #45332) and permitted by the Canadian Wildlife Service Migratory Bird Conservation Act Scientific Permits CA 0242 and CA 0308 and banding permits 10431V, 10431W, and 10901 from Environment and Climate Change Canada.

In 2009, we studied 21 Herring Gull breeding territories, all located either on the edge of, or within, the Ring-billed Gull colony (categorized as “near to Ring-billed Gulls”) (Table 1). In 2015, we selected two different natural “treatment” groups: (i) Herring Gulls nesting on the edges of/within the Ring-billed Gull colony (“near to Ring-billed Gulls”: 17 nests) and (ii) Herring Gulls nesting within their own colony at the southeast end of the island (“away from Ring-billed Gulls”: 10 nests) (Table 1). We marked all study breeding territories with nest stakes for identification. We calculated territory size for each as the product of the lengths of the territory measured during incubation in two dimensions (along north-south and east-west axes). In each case, length was the sum of the two distances along the axis from the nest to where study birds ceased to dive at investigators and landed, and markers were placed at these distances and adjusted if this changed on subsequent visits during incubation. For a field-based metric of territory density, we measured “nearest neighbor distances”, defined as linear measurements between nest cups of the focal territory and nearest active gull nest cup.

**Table 1:** Summary of study design.

Data	Aims	Method	Herring Gull Nests	Season	Years
Diet	Determine predatory specialism	Regurgitate pellet composition	21	Chick-rearing for Ring-billed Gulls	2009
		% Ring-billed Gulls in regurgitate pellets	27	Chick-rearing for Ring-billed Gulls	2015
		Number of Ring-billed Gull carcasses in Herring Gull territories	21, 27	Chick-rearing for Ring-billed Gulls	2009, 2015
Behavior	Estimate frequency of predation/ specialist behavior	In-person observations	37	Peak fledging period for Ring-billed Gulls	2015
		Remote video recordings	5	Peak fledging period for Ring-billed Gulls	2015
		Remote video recordings	8	Peak hatching period for Ring-billed Gulls	2016
	Evidence for hunting outside territory	Remote video recordings	N/A	Peak hatching period for Ring-billed Gulls	2016
Factors used in analysis		Territory size	21, 27	Incubating period for Herring Gulls	2009, 2015
		Nesting density (nearest neighbor distance)	21, 27	Incubating period for Herring Gulls	2009, 2015
		Shoreline access	21, 27	Incubating period for Herring Gulls	2009, 2015
		Treatment (near to/away from Ring-billed Gulls)	21, 27	Incubating period for Herring Gulls	2015

Overview of methods, sample sizes by year, and timing of samples (season, years) used to collect information on diet, behavior and factors/covariates for analyses. Yearly differences were not a focus of this study; protocols in subsequent years investigated questions that arose from data previously collected.

In 2009 and 2015, we recorded diet throughout the season by searching each territory for regurgitated Herring Gull pellets and prey carcasses (including all eaten remains: carcasses, fresh bones, large feathered remains) (Table 1) for at least five minutes on either nine (2009) or six (2015) different occasions between 24 May and 14 July. In 2015, new study nests were added up until 29 June, so the number of times a territory was searched during the study varied from 2–6 per nest (mean = 4.1). At first visit, visibly-old pellets and bird remains were destroyed and not recorded; for subsequent visits, carcasses were identified to species and age (adult/chick), and contents of pellets were recorded. In-person and video observations were used in 2015 and 2016 to estimate frequencies of predatory attacks, territorial aggression (aggressive interactions to or from study adults), feeding, scavenging and chick-provisioning by the breeding Herring Gulls (Table 1). We distinguished predatory attacks from territorial aggression if one of the following criteria was fulfilled: prey was chased further in to the breeding territory; if prey temporarily escaped it was pursued; an attempt was made to drown prey; or an attempt was made to eat prey. In 2015, 37 Herring Gull territories (both study and neighboring territories) were observed in person for between 31 and 120 min (totaling 164 territory-hours of observation). Observations were made from a temporary blind at least 7 m from any study nest, or twice from a boat anchored 30 m offshore, using binoculars and a 20× spotting-scope between 29 June and 15 July: this encompassed the peak fledging period for Ring-billed Gulls. During the same period, 4–6 remote trail cameras (Bushnell Trophy Cam Aggressor 119774C, Bushnell Trophy Cam HD 119576 [Overland Park, KS]) captured motion-triggered video of breeding Herring Gulls at 5 study territories during continuous periods (totaling 538 territory-hours of observation, range 40–222 h per territory). To supplement these data with observations encompassing the peak hatching period for Ring-billed Gulls (Table 1), 4–6 remote cameras (additional models: Browning Recon Force BTC-7FHD [Morgan, UT], Spypoint FORCE-11D [Swanton, VT]) were used in 2016 to record video at 8 study territories between 1 and 16 June during continuous 24–48 h periods (totaling 695 territory-hours of observation, range 29–238 h per territory).

In 2016, we also investigated the frequency with which gulls hunted outside their breeding territories (Table 1), a strategy used by predatory specialists [18,19]. Two infra-red trail cameras (Bushnell Trophy Cam HD) took combined motion-sensitive and time-lapse video (at least one 15 s video every 30 min) simultaneously either on the edge of the Ring-billed Gull colony next to (but not within) surrounding Herring Gull territories, or in the center of the Ring-billed Gull colony, at least 45 m from the nearest Herring Gull territory. Videos (87 and 68 observational-hours, respectively) were recorded on 6 d between 1 and 17 June (peak hatching period of Ring-billed Gulls) and examined for incidences of predation by Herring Gulls away from their breeding territories.

### Analyses

#### Diet and evidence for predatory specialists

In 2009, pellets were dissected and categorized as either fish, plant matter, terrestrial invertebrates, mammals, birds, or garbage; in 2015 only Ring-billed Gull remains (adult and chick) were quantified. Daily predatory impact was calculated as the number of birds killed per study Herring Gull pair per day, using the number of Ring-billed Gull carcasses found in Herring Gull territories, since carcasses have greater longevity than pellets [24,25].

For each study pair in 2009, we recorded the number of prey categories utilized, the most common prey item eaten, and the proportion of pellets in which this item was recorded. To investigate evidence for predatory specialists, we calculated both dietary niche width and dietary niche overlap [26,27]. We used the number of different prey categories recorded as an index of dietary niche width for each breeding territory. We used Bolnick et al.’s [26] standardized modification of Petraitis’ [28] likelihood approach to estimate dietary niche overlap as the probability of each pair’s diet being drawn randomly from the population distribution. This generated a P-value in each case to test whether an individual’s diet differed significantly from that of the population [26]. Since different prey have different energy densities, prior to this test we rescaled each pair’s pellet data according to relative energetic contributions. For each category, this was done by first estimating the proportion of each pellet that contained this prey category and then summing all these proportions to give the number of pellets of only that prey category. For the bird prey category, this sum was divided by 1.7 to compensate for over-representation of bird remains in regurgitated pellets [24,25] and render each pellet equivalent to a single meal. For each category, we then multiplied the energy density of prey (kJg^-1^ [15,29–31]) by average meal size [32] to determine the energy provided. Finally, we scaled energy provided to make it relative to other categories (by dividing by the average energy provided by all six categories) and multiplied it by the number of pellets of that prey category.

For each study nest in 2009 and in 2015 with sufficient data (4+ searches), we used carcass data to calculate daily bird prey consumption on each visit: dividing the number of carcasses recorded by the days since last search. We estimated that >0.5 carcasses/d would provide for the daily energetic demands of the two members of a breeding pair (1460 kJ/d [13]), estimating 2834 kJ per carcass by assuming all predated chicks were near fledging weight (400 g [33]), had an energy density of 10.9 kJ/g and only 65% of the carcass was digestible [29].

#### Behavioral observations and evidence for specialist behavior

We report the frequency of predatory attacks and aggression by Herring Gulls in 2015 and 2016 and contrast these between birds breeding in territories near to Ring-billed Gulls and those breeding away from Ring-billed Gulls. We also report predatory attacks by Herring Gulls away from their breeding territories detected in videos recorded in the center and edge of the Ring-billed Gull colony in 2016.

To screen for any specialized predatory behavior, behaviors used by adult Herring Gulls during each observed predatory attack were compiled from all territorial video footage and watch observations. Since behaviors occurred at different stages of an attack we categorized them into one of four time categories based on when they occurred: 0 min (immediately), 0–1 min, 1–5 min, and >5 min since initial attack. Behaviors were then contrasted with those reported as territorial aggression in the literature [31].

#### Seasonality and factors associated with predatory diets

Pellet and carcass data from 2009 and 2015 were plotted to examine seasonal trends in the proportion of Ring-billed Gulls within Herring Gull diets. Pellet and carcass data were also analyzed separately within each year using generalized linear models (GLMs) with binomial errors to determine whether territory size [large vs. small: ≥ 73.5 m^2^ (mean value) vs. < 73.5 m^2^], location [access to shoreline vs. no access to shoreline], and nearest neighbor distance affected the proportion of bird prey in dietary samples. In 2015, “treatment” (nesting near to Ring-billed Gulls vs. nesting in the small colony away from Ring-billed Gulls) and hatching date of the first egg from the focal Herring Gull nest (as a Julian date; “Hatching Date”) were additional predictors.

All GLM model selection was implemented in the R package *MuMIn* [34] to determine the most parsimonious model reduction [35] from the maximal model (main effects for all predictors). We tested GLMs for overdispersion (c-hat > 1) and corrected for this by ranking models by QAICc (instead of AICc) in the R package *AICcmodavg* [36]. Where competing models were within 2 QAICc of the top-ranked model, model averaging was used to determine the relative importance of each predictor across these competing models [35]. Number of model parameters (K), difference in ranking criteria from top model (ΔQAICc) and proportional likelihood of the model (QAICc weights: relative likelihood of the model divided by the likelihood sum of all models) are reported [35].

All means are reported with ± 1 SE and medians with {lower quartile, upper quartile}, unless otherwise indicated.

## Results

### Diet and evidence for predatory specialists

Waterbird remains were found in 33% of all 265 pellets analyzed in 2009, garbage and other human foodstuffs in 24%, fish in 18%, plant material in 13%, mammal remains in 6.5% and insects in 2.6% (2.9% of items were unidenitfiable). 51% of waterbird prey was Ring-billed Gull chick, 36% Ring-billed Gull adult, 2% Caspian Tern chick and 11% was unidentifiable. At any one territory, the most common prey item occurred at most in only 38 {30, 46} % of regurgitated pellets. Dietary niche width was broad: on average 4 {3, 5} of the 6 different prey categories were exploited by each study pair (range: 2–6 categories). Dietary niche overlap was common: only 25% (5/20) of pairs differed significantly in their use of prey categories (P<0.05) from the population average. Of those that differed, only 1 ate more birds and all still exploited prey from many different categories (niche width = 4–5 in all cases). Daily predatory impact (Ring-billed Gulls killed per Herring Gull pair per d) inferred from the number of Ring-billed Gull carcasses found in Herring Gull territories was 0.41 in 2009 and 0.10 in 2015.

**Fig 1:**
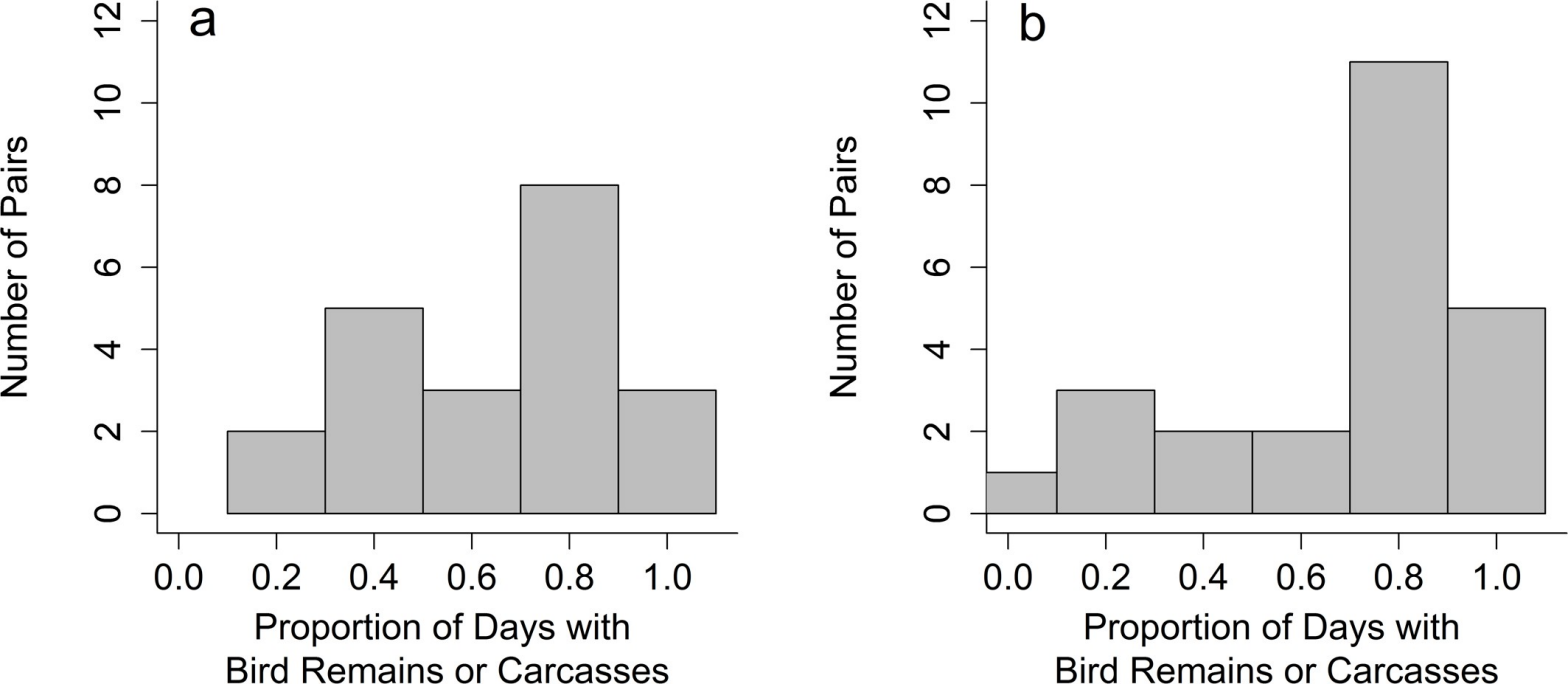
Variation in exploitation of Ring-billed Gulls as prey by Herring Gulls. Frequency of exploitation of Ring-billed Gull prey (RBGU) by individual Herring Gull pairs in (a) 2009 and (b) 2015, as indicated by either remains found in pellets or carcasses in territories. Data are only shown for pairs in which four or more days of dietary records were available.

Individual Herring Gulls varied widely in their predation of birds (Fig 1) and bird remains were found in all but one of the 48 study breeding territories (98%, Fig 1). For 27% (12/45) of study pairs the average predation rate across the season was >0.5 kills/d (sufficient to meet energetic demands) but no study pairs achieved this level during every sampling interval (see *Seasonality and factors associated with predatory diets* below).

### Behavioral observations and evidence for specialist behavior

Twenty-two attacks of Herring Gulls on Ring-billed Gulls were observed during 164 territory-hours of in-person watches within the peak fledging period for Ring-billed Gulls (July 2015), and all took place within Herring Gull breeding territories (overall attack rate: 0.13 attacks/h/territory). However, only 36% of attacks resulted in a kill. These predatory attacks by breeding Herring Gulls were four-times more frequent at Herring Gull territories near to the Ring-billed Gull colony than those away from Ring-billed Gulls (0.24 vs 0.08 attacks/h/territory). This relationship followed that for territorial aggression (0.31 vs 0.07 aggressive interactions/h/territory, respectively). Attacks were less commonly observed on remote video recordings at Herring Gull territories: only 10 attacks were observed during 537 h of footage over the same period (July 2015). This is likely because of limitations in the field of view of cameras when vegetation and neighbors sometimes caused unexpected change to territory boundaries. No predatory attacks were ever observed in 695 h of remote video footage at Herring Gull territories during the peak hatching period for Ring-billed Gulls (May 2016). Also during this time period, no Herring Gulls were observed hunting outside their breeding territories, either within the Ring-billed Gull colony or along its edge (155 h of time-lapse video recordings in 2016).

**Fig 2:**
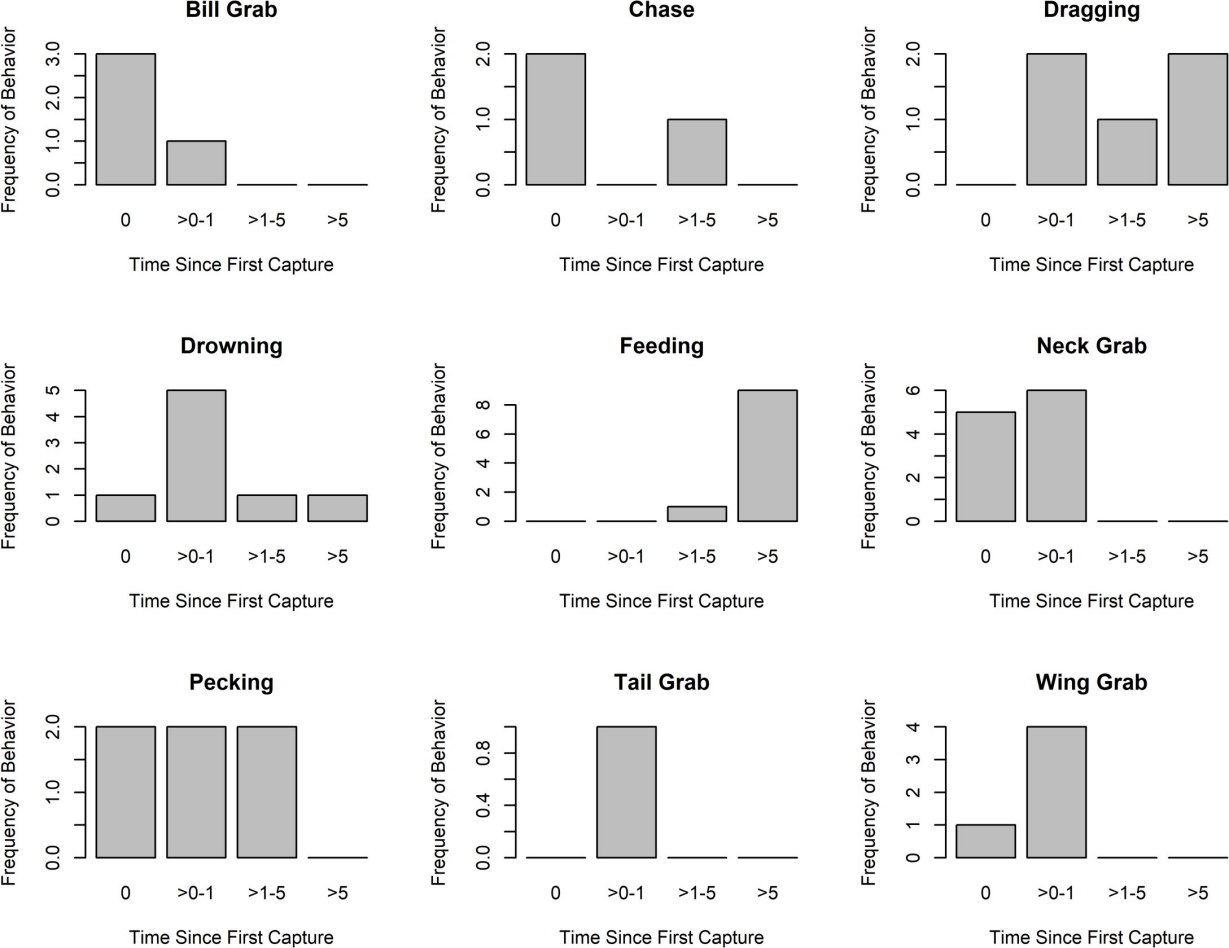
Timing of behaviors used during predatory attacks by Herring Gulls. Timings (minutes since initiation of attack) are given for all different behaviors across all observed attacks (both successful and failed) observed by Herring Gulls upon Ring-billed Gull chicks.

On average, attacks by Herring Gulls on Ring-billed Gulls lasted 3.9 ± 3.3 min (max = 22 min). During predation events, Herring Gulls engaged in the following behaviors: chasing; bill-, neck-, wing- and tail-grabbing; dragging; drowning; and pecking (of head or belly) (Fig 2). Although these events were clearly classified as attacks, these behaviors are not specialist behaviors as they are used by Herring Gulls during territorial disputes [31]. Within the first two minutes of an attack, Herring Gulls seized Ring-billed Gull chicks by their neck or bill (Fig 2) and, if within 1 m of the shoreline, attempted to drown them. Neck Grabs were by far the most common behavior in the first minute of an attack (Fig 2), and once a good neck hold was established the prey would be dragged further into the territory to be drowned or pecked. Wing and Tail Grabs were mainly the result of losing grip of the prey and attempting to re-catch it after a brief chase (Fig 2). Pecking of the head and stomach occurred up to 5 min from initiation of the attack but was then replaced by active feeding (Fig 2).

### Seasonality and factors associated with predatory diets

**Fig 3:**
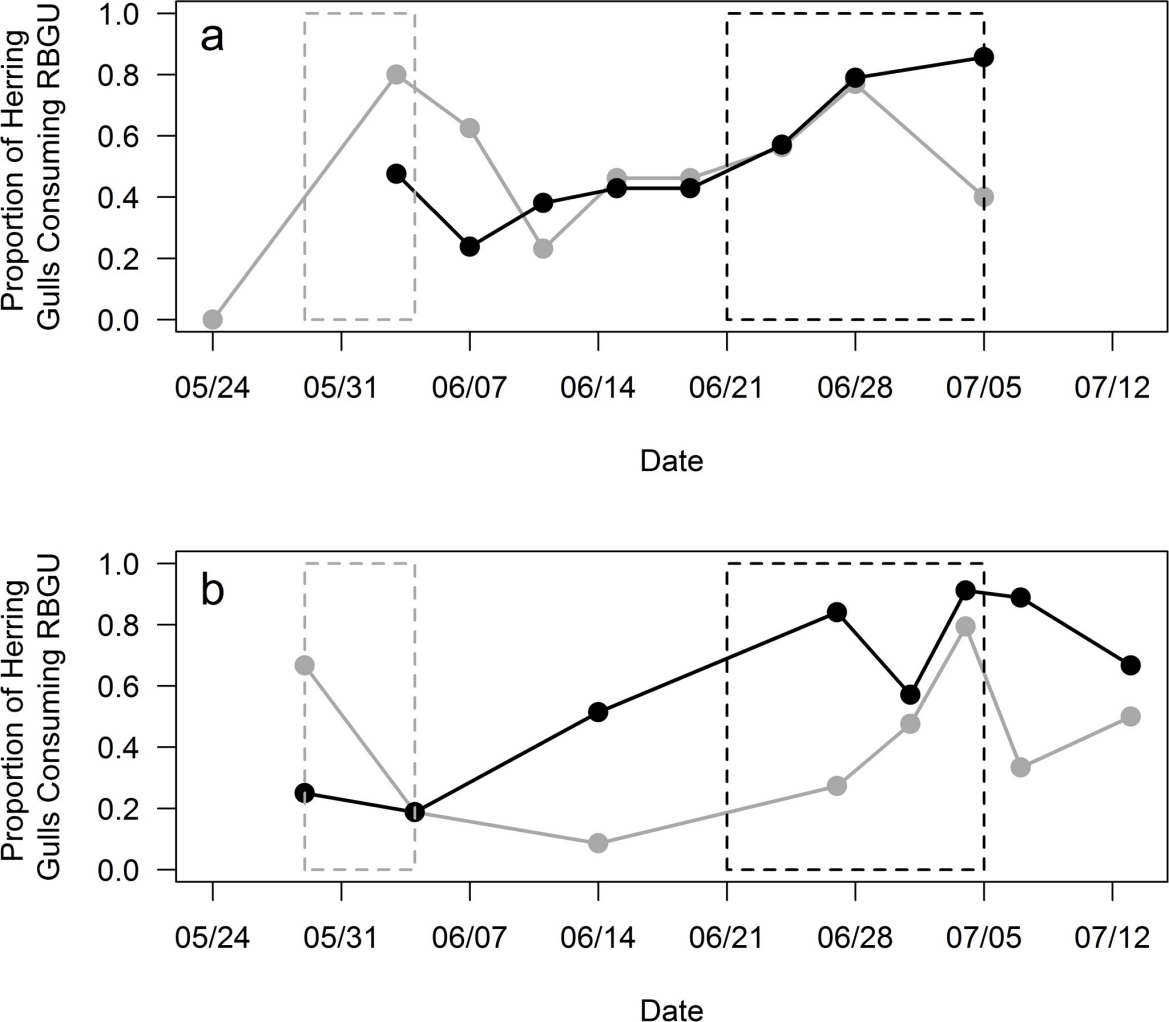
Seasonal exploitation of bird prey by Herring Gulls in two years of study. Proportion of studied Herring Gull pairs consuming Ring-billed Gull chicks (RBGU), as indicated by remains found in pellets (gray) or carcasses (black), throughout the study period in (a) 2009 and (b) 2015. Peak hatching (dashed gray box) and peak fledging period of Ring-billed Gull chicks (dashed black box) are indicated. Early in the season, when Ring-billed Gull chicks are small enough to swallow whole, estimated exploitation of RBGU is higher from pellet analyses.

Pellet analyses indicated that more breeding Herring Gulls consumed Ring-billed Gull during peak hatching and peak fledging periods of Ring-billed Gulls (Fig 3). Carcass data, however, only indicated a marked increase during the peak fledging period (Fig 3).

**Fig 4:**
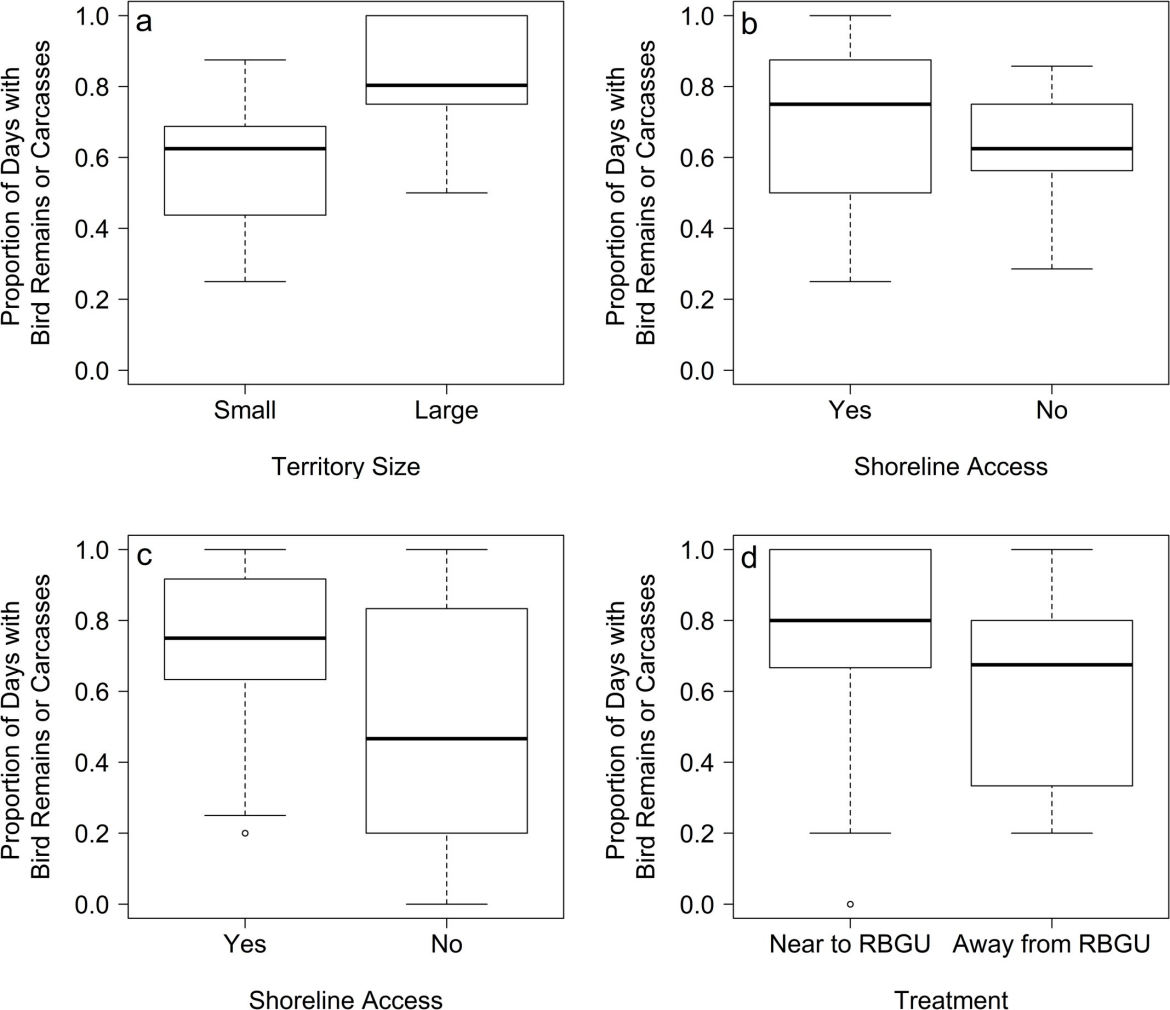
Factors associated with predatory diets of Herring Gulls. Boxplot of most important factors predicting diet of Herring Gulls during the two years of intensive study: (a) territory size and (b) shoreline access in 2009, and (c) shoreline access and (d) treatment in 2015. Data are frequencies with which Ring-billed Gull chick (RBGU) carcasses or remains within pellets were found in Herring Gull territories. Note: all territories studied in 2009 were on the edge of/ or within the ring-billed colony and thus it was not possible to assess treatment effects in this year.

In 2009, when all Herring Gull territories studied were close to Ring-billed Gull nests (i.e. there was no “treatment”), shoreline access and territory size were most important in determining the frequency with which both Ring-billed Gull remains were found in Herring Gull pellets and Ring-billed Gull carcasses were found in Herring Gull territories (Tables 2A and 3A and Fig 4A and 4B). In 2015, pellets containing Ring-billed Gull remains were slightly more common in Herring Gull territories located near to Ring-billed Gulls (0.44 ± 0.07) than in territories away from Ring-billed Gulls (0.35 ± 0.07) [“Treatment”]. However, this effect was secondary to having a territory with access to the shoreline (Table 2B, Relative Importances: 1.00 [Shoreline Access] vs 0.70 [Treatment]). These same relationships were stronger for the number of carcasses found in Herring Gull territories (0.67 ± 0.07 near to Ring-billed Gull colony vs. 0.51 ± 0.11 away from Ring-billed Gull colony; Table 3B, Relative Importances: 1.00 [Shoreline Access] vs 0.65 [Treatment]). Thus, overall consumption of Ring-billed Gulls by Herring Gulls was higher among territories with shoreline access (Fig 4C) close to the Ring-billed Gull colony (Fig 4D).

**Table 2:** Highest-ranked GLM models predicting presence of bird remains in Herring Gull pellets.

(a) 2009					(b) 2015				
Parameters	K	QAIC_c_	ΔQAIC_c_	QAIC_c_ Weight	Parameters	K	QAIC_c_	ΔQAIC_c_	QAIC_c_ Weight
**Shoreline Access**	3	60.9	0.00	0.39	**Shoreline Access**	3	56.9	0.00	0.27
**Shoreline Access, Territory Size**	4	62.3	1.40	0.20	**Shoreline Access, Treatment**	4	57.4	1.56	0.21
**Null Model**	2	62.8	1.89	0.15	**Shoreline Access, Nearest Neighbor**	4	58.9	2.02	0.10
**Shoreline Access, Nearest Neighbor**	4	63.8	2.97	0.09	**Shoreline Access, Territory Size**	4	59.2	2.36	0.08
**Territory Size**	3	64.3	3.39	0.07	**Shoreline Access, Hatching Date**	4	59.6	2.70	0.07

Five highest-ranked GLMs from model selection of factors affecting whether Herring Gull pellets containing Ring-billed Gull remains in (a) 2009 and (b) 2015. Null Model–indicates intercept only for this parameter. Relative Importance of each predictor was (a): Shoreline Access = 1.00, Territory Size = 0.40; (b) Shoreline Access = 1.00, Treatment = 0.70.

**Table 3:** Highest-ranked GLM models predicting presence of bird carcasses in Herring Gull territories.

(a) 2009					(b) 2015				
Parameters	K	QAIC_c_	ΔQAIC_c_	QAIC_c_ Weight	Parameters	K	QAIC_c_	ΔQAIC_c_	QAIC_c_ Weight
**Territory Size**	3	46.3	0.00	0.59	**Shoreline Access, Treatment**	4	50.8	0.00	0.22
**Shoreline Access, Territory Size**	4	48.4	2.16	0.20	**Shoreline Access**	3	51.1	0.25	0.19
**Territory Size, Nearest Neighbor**	4	48.8	2.56	0.16	**Shoreline Access, Nearest Neighbor**	4	52.2	1.37	0.11
**Shoreline Access, Territory Size, Nearest Neighbor**	5	51.1	4.84	0.05	**Shoreline Access, Treatment, Hatching Date**	5	52.3	1.53	0.08
					**Shoreline Access, Nearest Neighbor, Hatching Date**	5	52.8	1.98	0.08

Top-ranked GLMs from model selection of factors affecting whether Ring-billed Gull carcasses were found in Herring Gull territories in (a) 2009 and (b) 2015. For (b), the Relative Importance of each predictor was: Shoreline Access = 1.00, Treatment = 0.65, Hatching Date = 0.58, and Nearest Neighbor = 0.30.

## Discussion

Large gulls and skuas are well known for exhibiting individual dietary specialization [7] and predation of birds is widely reported as a specialist strategy employed by a minority of individuals in the population [14,15,17,32]. However, our analyses of diet and behavior revealed that predation of Ring-billed Gulls (the predominant bird prey) by Herring Gulls was opportunistic at Gull Island and not a specialist strategy. Dietary data indicated that Ring-billed Gulls, by far the most numerous nesting waterbird in our study area, were widely exploited as prey, utilized by nearly all Herring Gulls in correspondence with their seasonal availability (Fig 3) and the proximity of both nests and roosts (Fig 4). Herring Gull pairs maintained broad dietary niches and limited any one prey type to a maximum of one-third of their diet. Only one pair exploited birds as prey significantly more than normal and even those individuals were not specialists as they also utilized many other prey types. Predation at individual Herring Gull territories occurred too infrequently to reliably meet all the energetic demands of a breeding pair throughout the season, was only observed within territories, and did not require specialized hunting behaviors (behaviors observed were those used during intraspecific territorial disputes, c.f. Fig 2 and [31]). The predatory attacks we did observe were also largely unsuccessful (only 36% of attacks observed resulted in death of the prey) and below success rates of specialist predatory gulls (50%-75%) [10,37] (but more successful than reported previously for opportunistic gulls (15%-22%) [38,39], probably because all chicks were attacked within herring gull breeding territories with no parent to defend them). Thus, although predating other birds comprised an important proportion of their diet (33% on average), Herring Gulls at Gull Island were non-specialists that opportunistically predated birds.

Our results contrast with the only other study in the region (Rogers City, western Lake Huron), where Herring Gulls bred within a colony of ~10,000 pairs of Ring-billed Gulls [11]. Of the ~30 Herring Gulls with territories on the perimeter of that Ring-billed Gull colony, only 25% fed on Ring-billed Gulls in one year of study but 71% predated gulls in the following year [11]. The authors attribute much of this variation to small sample sizes (n < 6) [11], but it is likely that opportunism is also occurring, with birds switching to predatory diets during food shortfall as in other studies [10,12]. However, many Herring Gulls at Rogers City did appear to be predatory-specialists, hunting outside their territories and having much larger territories (up to 120 m diameter [11]) than in our study (maximum of 30 m), consistent with the elongation of breeding territories into feeding territories by specialists that has been reported elsewhere (e.g. [22]). These birds also had a greater daily predatory impact (2.7 Ring-billed Gulls killed per pair per day) than in our study (0.41 in 2009, 0.10 in 2015). It should be noted that these perimeter-nesting specialists comprised only 3–4% of the Rogers City Herring Gull breeding colony. By contrast, at Gull Island practically all Herring Gulls consumed bird prey and thus predation by non-specialists is likely much more important. A similar but less extreme situation to Gull Island has been reported for skuas: of all the birds predated by Great Skuas *Stercorarius skua* at a colony in Shetland, UK, > 70% were taken by non-specialists [15].

Exploitation of bird prey by our non-specialist Herring Gulls was associated with access to shoreline, proximity to nesting Ring-billed Gulls and, to a lesser extent, breeding territory size. These factors likely lead to increased frequency of Ring-billed Gull chicks (the most common bird prey exploited) straying into Herring Gull breeding territories. Having access to the shoreline within their territory likely increased encounters with fledgling Ring-billed Gull chicks that crèched on the beach in large numbers often without adults present (e.g. [40]), but also increased the chances of making a successful kill (drowning prey was a common predation tactic within the first minute of an attack). Although shoreline access was a strong predictor of predatory diets whether determined by pellets or carcasses, the two other factors were stronger predictors of the presence of eaten Ring-billed Gull carcasses than of bird remains in regurgitated Herring Gull pellets (compare Tables 2 and 3). This is presumably because carcasses are more conspicuous than pellets, which may quickly degrade [24,25]. These results suggest that non-specialist Herring Gulls exploit bird prey opportunistically and that easy access to vulnerable life-stages (such as undefended chicks) and situations that promote the success of attacks are critical factors promoting the exploitation of bird prey. Previous studies have reported that time constraints and predictability may be more important for gulls and skuas than energetic value of prey [13,23,41], with birds choosing diets that allow prolonged attendance at nests to defend against intruders [13]. Since predation of birds at Gull Island occurred exclusively within Herring Gull nesting territories, it is not surprising that nearly all study pairs opportunistically consumed birds and to a greater extent than at other colonies [11]. This opportunism agrees with interspecific and between-colony studies that show predation levels decline with limited access to bird prey [42] and greater availability of alternative prey [7].

Waterbirds often nest at high densities within large, mixed-species, breeding colonies [43]. For many waterbird species, such colonies generally support large proportions of their overall population [44,45]. Thus, even a low level of opportunistic predation by non-specialist gulls or skuas that also breed there could have a greater impact on these populations than that of a few specialist predatory individuals [42]. In our study of ~20% of the Herring Gulls breeding at Gull Island, as many as 292 Ring-billed Gulls were found killed inside Herring Gull territories during the chick-rearing stage (based on carcasses recovered in 2009). This equates to ~1,500 killed colony-wide on an annual basis and suggests that opportunistic predation by Herring Gulls affects ~5% of all 30,000 Ring-billed Gull nests. Although this level of predation may be low enough to be sustainable in this colony, our results indicate that where Herring Gulls numbers are proportionally higher, for example at smaller Ring-billed Gull colonies, this opportunistic predation could have major impacts on breeding success of prey species. Compared with predatory specialist Herring Gulls in the same region [11], individuals that predated birds opportunistically at Gull Island killed six times fewer birds (0.1–0.4 per day, on average) but were over 20 times more numerous (98% of the population versus 4%), implying a greater overall predation pressure. Thus, the potential impact of opportunistic predation by non-specialists should not be overlooked.

Although removing specialist gulls is strongly advocated as a cost-effective management strategy [16,17], opportunistic predation by large gulls cannot be effectively managed using the same techniques. This is because removed individuals would be quickly replaced by conspecifics that require no specialized behavior. Large-scale culling would be required which has been shown to be expensive, often ineffectual and socially undesirable [17,46–48]. It is therefore vital to confirm the presence of specialists before undertaking lethal control. Although presence of bird remains on breeding territories or in regurgitated pellets have been successfully used in the past to identify specialists (e.g. [17,46]), in our study these field signs were indicative of non-specialist gulls that ate birds opportunistically. Consequently, we advocate investigating both diet and behavior of potential specialists prior to implementing lethal management. Our results suggest that, if purely opportunistic, predation could be minimized through the creation of safe crѐching or gathering areas for the waterbird species targeted by gulls, for example by use of exclusion-style fencing [49]. Such approaches would need to be tested and refined, but our study indicates that opportunistic predation occurs only within Herring Gull territories implying that physical barriers that prevent birds wandering into gull breeding territories could reduce the predatory impacts of large gulls at mixed-species breeding colonies.
